# Importance of Hepatic Copper Content as a Single Diagnostic Criterion for Wilson’s Disease: A Case Report

**DOI:** 10.1155/crhe/7940683

**Published:** 2026-11-14

**Authors:** Hoda Haghshenas, Mehdi Karimi, Solmaz Hassni Fard Katiraie, Maryam Ataollahi, Fereshteh Karbasian

**Affiliations:** ^1^ Faculty of Medicine Jahrom University of Medical Sciences (JUMS) Jahrom Iran; ^2^ Faculty of Medicine Bogomolets National Medical University (NMU) Kyiv Ukraine; ^3^ Department of Medical Genetics Shiraz University of Medical Sciences Shiraz Iran sums.ac.ir; ^4^ Shiraz Transplant Center Abu-Ali Sina Hospital Shiraz University of Medical Sciences Shiraz Iran sums.ac.ir; ^5^ Department of Pediatric Gastroenterology and Hepatology Ali-Asghar Children’s Hospital Iran University of Medical Sciences Tehran Iran iums.ac.ir

**Keywords:** *ATP7B* mutation, case report, hepatic copper, hepatitis, pediatric, Wilson’s disease

## Abstract

**Background:**

Wilson’s disease (WD) is a rare autosomal recessive disorder caused by mutations in the *ATP7B* gene, resulting in impaired copper metabolism and toxic accumulation in the liver. In pediatric patients, diagnosis may be particularly challenging due to the absence of classic manifestations.

**Case Presentation:**

We report the case of a 4‐year‐old girl (16 kg) who was asymptomatic but had persistently elevated liver enzymes detected during routine evaluation from the age of one year. She had no clinical, neurological, or ophthalmologic signs suggestive of WD. Comprehensive laboratory investigations, including viral and autoimmune markers, were unremarkable. Given the persistent transaminase elevation and lack of an alternative diagnosis, a liver biopsy was performed. Histopathological examination demonstrated features consistent with chronic active hepatitis, and quantitative analysis revealed markedly elevated hepatic copper content (1605 μg/g dry weight). Subsequent whole‐exome sequencing identified a homozygous *ATP7B* mutation, confirming the diagnosis of WD.

**Management and Outcome:**

Treatment with zinc and trientine was initiated, resulting in normalization of liver enzyme levels within 3 months. Sustained biochemical remission was maintained throughout the 1‐year follow‐up period.

**Key Clinical Message:**

This case underscores the pivotal diagnostic role of hepatic copper quantification in children with atypical or clinically silent presentations of WD. Although serum ceruloplasmin and urinary copper excretion are widely used screening tools, they may lack sensitivity and specificity in young children. Hepatic copper measurement remains a definitive diagnostic criterion and should be strongly considered when clinical and laboratory findings are inconclusive, facilitating early diagnosis and timely initiation of therapy.

## 1. Introduction

Wilson’s disease (WD) is a rare autosomal recessive disorder caused by mutations in the *ATP7B* gene, resulting in impaired copper excretion and toxic accumulation of copper, primarily in the liver [1, 2]. In pediatric patients, hepatic involvement is often the earliest manifestation; however, diagnosis remains challenging due to variable clinical presentations and the frequent absence of classical features such as Kayser–Fleischer rings or neurological symptoms [3]. Standard diagnostic approaches rely on a combination of clinical findings, biochemical markers, and scoring systems, which may lack sensitivity in young or presymptomatic children [1, 2].

Hepatic copper quantification obtained through liver biopsy is a well‐established diagnostic method, with levels exceeding 250 µg/g dry weight considered highly suggestive of WD [4]. Despite its diagnostic value, this method is often underutilized due to its invasive nature, particularly when other diagnostic parameters are inconclusive. Consequently, the diagnosis of WD may be delayed in children presenting with isolated and persistent elevations of liver enzymes [3, 4].

We report the case of a 4‐year‐old girl with persistently elevated liver enzymes and no clinical, ophthalmologic, or neurological signs of WD. In the absence of supportive biochemical or clinical findings, the diagnosis was confirmed solely by quantification of hepatic copper. This case highlights the importance of hepatic copper content as a decisive diagnostic criterion for WD, emphasizing its role in early diagnosis and timely initiation of treatment in pediatric patients.

## 2. Case Presentation

### 2.1. Case Information

A 4‐year‐old girl, weighing 16 kg, born to consanguineous parents, was referred to the pediatric gastroenterology department for evaluation of persistently elevated liver enzyme levels. These abnormalities were initially detected during routine annual health evaluations at the age of 1 year and remained consistently elevated on subsequent follow‐up assessments.

The patient was born at term following an uncomplicated pregnancy and delivery, with no significant past medical history. She remained clinically asymptomatic despite the persistent biochemical abnormalities. There was no history of previous illnesses, surgical procedures, or exposure to herbal or indigenous medications. The child was the only offspring of healthy parents, and there was no family history of liver disease or similar conditions. No exposure to hepatotoxic drugs or environmental toxins was reported.

### 2.2. Clinical Assessment

A comprehensive review of systems was unremarkable. The patient had no gastrointestinal symptoms, including abdominal pain, gastrointestinal bleeding, vomiting, or changes in bowel habits. Neurological review revealed no history of seizures, altered mental status, or developmental delay. There were no dermatological manifestations, including jaundice, rash, petechiae, or ecchymosis. Additionally, there was no history of arthralgia, arthritis, fever, weight loss, or fatigue.

On physical examination, the patient appeared well and in no distress. She was afebrile, conscious, and alert, with stable vital signs. There were no stigmata of chronic liver disease, including jaundice or palmar erythema. Abdominal examination revealed a soft, nontender abdomen with no hepatomegaly, splenomegaly, or ascites. Examination of the extremities showed no edema, clubbing, or bleeding manifestations. Ophthalmologic evaluation, including slit‐lamp examination for Kayser–Fleischer rings, was normal. Neurological examination revealed no abnormalities.

### 2.3. Diagnostic Investigations

Laboratory investigations are summarized in Table 1. Autoimmune and viral markers were negative. Hepatic copper concentration was 1605 μg/g. Abdominopelvic ultrasonography and echocardiography results were normal.

**TABLE 1:** Laboratory findings of the patient with Wilson’s disease.

Values	1‐year‐old	Time of diagnosis	Follow‐up	Reference range
Total leukocyte count	6.93	—	6.6	5.5–15.5

Hb (g/dL)	12.60	—	13.6	11–14

Platelet	312	—	323	200–490

Mg (mg/dL)	2.0	—	2.1	1.6–2.5

Zn (μg/dL)	90	—	90	64–110

CU (μg/dL)	—	—	72	80–190

Urea (mg/dL)	—	—	28	15–36

Creatinine (mg/dL)	—	—	0.65	0.60–1.30

AST (IU/L)	65	156	57	Children, up to 45

ALT (IU/L)	19	189	33	Children, up to 40

ALP (IU/L)	—	880	830	180–1200

Total bilirubin (mg/dL)	—	0.49	0.39	0.1–1.2

Fasting blood sugar (mg/dL)	—	89	90	74–127

Triglycerides (mg/dL)	—	118	—	Desirable: < 200

Total cholesterol (mg/dL)	—	197	—	Desirable: < 200

HDL cholesterol (mg/dL)	—	63	—	> 35

LDL cholesterol (mg/dL)	—	110	—	Desirable: < 130

Direct bilirubin (mg/dL)	—	0.17	0.15	UP to 0.3

PT (sec)	—	13.0	13.0	11–13.5

PTT (sec)	—	33	34	30–44

INR	—	1	1	

CPK total (U/L)	—	78	—	24–229

LDH (U/L)	—	424	—	< 850

Total protein (g/dL)	—	6.7	6.4	5.7–8

Albumin (g/dL)	—	5.0	4.6	3.5–5.2

Random urine creatinine (mg/dL)	—	—	126	16–327

Urine volume (mL/24 h)	—	300	400	600–700

Urine copper (μg/24 h)	—	28	48	Up to 90

Gamma glutamyl transferase (U/L)	—	29	16	3–32

Ceruloplasmin (mg/dL)	—	24.2	—	20–60

IgA (mg/dL)	—	143	—	19–220

ASCA (Au/mL)	—	< 3.0	—	Negative: up to 12 Borderline: 12–18 Positive: more than 18

ANA (U/mL)	—	6.27	—	Negative: < 12 Equivocal: 12–18 Positive: > 18

Anti‐TTG (G) (Au/mL)	—	7.6	—	Negative: up to 12 Borderline: 12–18 Positive: more than 18

Anti‐TTG (A) (Au/mL)	—	3.5	—	Negative: up to 12 Borderline: 12–18 Positive: more than 18

LKM (Ab) (RU/mL)	—	1.3	—	Up to 20

Ferritin (mg/L)	17.3		16.0	20–110

Free T4 (ng/dL)	1.2	0.993	—	0.7–1.8

TSH (μIU/mL)	3.00	1.55	—	0.4–5

HIV Ab (I + II)	—	Nonreactive	—	Nonreactive

HBs Ag	—	Nonreactive	—	Up to 0.89: nonreactive 1.28–2.52: low reactive 2.6–9.0: reactive

HCV–Ab	—	Nonreactive	—	Nonreactive < 1 Reactive > 1

Urine analysis		—		—

Color	Yellow	—	Yellow	—

Appearance	Semiclear	—	Clear	—

Specific gravity	1.020	—	1.028	—

pH	5	—	5	—

Protein	Negative	—	Negative	—

Glucose	Negative	—	Negative	—

Bilirubin		—	Negative	—

*Note:* Hb: hemoglobin, Mg: magnesium, Zn: zinc, Cu: copper, AST: aspartate aminotransferase, ALT: alanine aminotransferase, ALP: alkaline phosphatase, CPK: creatine phosphokinase, LDH: lactate dehydrogenase, IgA: immunoglobulin A, ANA: antinuclear antibody, Anti‐TTG (G): anti–tissue transglutaminase antibody (IgG), Anti‐TTG (A): anti–tissue transglutaminase antibody (IgA), LKM Ab: liver kidney microsomal antibody, T4: thyroxine, HIV Ab: human immunodeficiency virus antibody, HBs Ag: hepatitis B surface antigen, HCV Ab: hepatitis C virus antibody.

Abbreviations: ASCA, anti–*Saccharomyces cerevisiae* antibody; BUN, blood urea nitrogen; GGT, gamma‐glutamyl transferase; HDL, high‐density lipoprotein; INR, international normalized ratio; LDL, low‐density lipoprotein; PT, prothrombin time; PTT, partial thromboplastin time; TSH, thyroid‐stimulating hormone.

Whole‐exome sequencing identified a homozygous mutation in the *ATP7B* gene, with both parents confirmed as carriers. The liver biopsy revealed a chronic active hepatitis pattern with interface activity, steatosis, and vacuolization of hepatocyte nuclei, consistent with WD.

### 2.4. Management and Follow‐Up

The patient was started on zinc therapy at a dose of 25 mg twice daily, alongside trientine 20 mg/kg twice daily. Liver enzyme levels returned to the normal range within 3 months of treatment initiation. After a one‐year follow‐up period, the patient demonstrated significant improvements in laboratory test results, as summarized in Table 1.

## 3. Discussion

WD remains a diagnostic challenge in pediatric patients, particularly in early childhood, when classical clinical and biochemical features may be absent or incomplete [3]. The present case illustrates a presentation of WD in a young, asymptomatic child with isolated and persistent elevation of liver enzymes, in whom the diagnosis was ultimately established through markedly elevated hepatic copper content. This finding underscores the critical diagnostic value of hepatic copper quantification, especially when other commonly used parameters fail to provide conclusive evidence.

In children, hepatic involvement is often the earliest manifestation of WD, yet the disease may remain clinically silent for years. Serum ceruloplasmin levels and 24‐h urinary copper excretion, although widely used, have limited sensitivity and specificity in pediatric populations and should therefore be interpreted alongside other diagnostic parameters. Ceruloplasmin may be normal in up to 40% of affected children, particularly in presymptomatic cases or during inflammatory states, while urinary copper excretion can overlap with other chronic liver diseases [5]. Hepatic copper quantification is particularly valuable when conventional findings are inconclusive. Recent studies also highlight the potential roles of copper‐mediated cuproptosis and iron‐related ferroptosis in WD pathophysiology [6].

In the present case, the absence of Kayser–Fleischer rings, neurological symptoms, and overt clinical signs could have delayed diagnosis if reliance had been placed solely on noninvasive markers.

Hepatic copper quantification remains an important diagnostic tool in the evaluation WD, with levels exceeding 250 µg/g dry weight considered highly suggestive of the disease [7]. The exceptionally high hepatic copper concentration observed in this patient strongly supported the diagnosis, even in the absence of classical manifestations. Histopathological findings consistent with chronic active hepatitis further strengthened the diagnostic conclusion [8]. These results are consistent with previous studies emphasizing that liver biopsy retains particular importance in young children and in cases with ambiguous biochemical profiles.

Identification of a homozygous ATP7B mutation by whole‐exome sequencing provided definitive genetic confirmation and enabled family screening, revealing heterozygous carrier status in both parents [9]. Although genetic testing is increasingly accessible, it may not always be readily available or rapidly interpretable in all clinical settings. Therefore, hepatic copper measurement remains pivotal, particularly in resource‐limited settings or when genetic findings are inconclusive.

Early diagnosis is essential, as prompt initiation of chelation and zinc therapy can prevent disease progression and irreversible organ damage [10, 11]. The favorable biochemical response observed in this patient following treatment highlights the effectiveness of early therapeutic intervention and reinforces the importance of maintaining a high index of suspicion for WD in children with unexplained, persistent liver enzyme abnormalities.

Despite its strengths, this report is limited by its nature as a single case study, which restricts generalizability. Nevertheless, it adds to the growing body of evidence that quantification of hepatic copper should not be overlooked, even as a standalone diagnostic criterion, in pediatric patients with atypical or silent presentations of WD.

## 4. Conclusion

In conclusion, this case emphasizes that WD should be considered in any child with persistent, unexplained elevations in liver enzymes, regardless of the absence of classical clinical signs. Hepatic copper quantification remains a decisive and reliable diagnostic tool and should be utilized early when standard diagnostic parameters are inconclusive.

## Author Contributions

Case presentation, writing, and supervision: Hoda Haghshenas, Fereshteh Karbasian, Solmaz Hassni Fard Katiraie, Maryam Ataollahi, and Mehdi Karimi.

Conceptualization and critical review and editing: Hoda Haghshenas and Mehdi Karimi.

All authors contributed to the interpretation of the clinical findings and critically reviewed the manuscript for important intellectual content.

## Funding

The authors have nothing to report.

## Disclosure

All authors approved the final version of the manuscript and agree to be accountable for all aspects of the work.

## Ethics Statement

Written informed consent for publication of this case report and the accompanying clinical images was obtained from the patient’s parents/legal guardians. Ethical approval was not required according to institutional policy for the publication of a single anonymized case report. This case report was prepared in accordance with the CARE reporting guideline.

## Consent

Written informed consent was obtained from the patient’s parents/legal guardians for the publication of this case report, including the publication of clinical details and any accompanying images.

## Conflicts of Interest

The authors declare no conflicts of interest.

## Supporting Information

Additional supporting information can be found online in the Supporting Information section.

## Supporting information


**Supporting Information** CARE checklist.

## Data Availability

All relevant data are included in this published article. Further data are not publicly available due to patient privacy considerations but may be obtained from the corresponding author upon reasonable request.
